# Constraint-Based Modeling and Kinetic Analysis of the Smad Dependent TGF-*β* Signaling Pathway

**DOI:** 10.1371/journal.pone.0000936

**Published:** 2007-09-26

**Authors:** Zhike Zi, Edda Klipp

**Affiliations:** 1 Computational Systems Biology, Max Planck Institute for Molecular Genetics, Berlin, Germany; 2 Theoretical Biophysics, Humboldt University Berlin, Institute for Biology, Berlin, Germany; IBM Thomas J. Watson Research Center, United States of America

## Abstract

**Background:**

Investigation of dynamics and regulation of the TGF-β signaling pathway is central to the understanding of complex cellular processes such as growth, apoptosis, and differentiation. In this study, we aim at using systems biology approach to provide dynamic analysis on this pathway.

**Methodology/Principal Findings:**

We proposed a constraint-based modeling method to build a comprehensive mathematical model for the Smad dependent TGF-β signaling pathway by fitting the experimental data and incorporating the qualitative constraints from the experimental analysis. The performance of the model generated by constraint-based modeling method is significantly improved compared to the model obtained by only fitting the quantitative data. The model agrees well with the experimental analysis of TGF-β pathway, such as the time course of nuclear phosphorylated Smad, the subcellular location of Smad and signal response of Smad phosphorylation to different doses of TGF-β.

**Conclusions/Significance:**

The simulation results indicate that the signal response to TGF-β is regulated by the balance between clathrin dependent endocytosis and non-clathrin mediated endocytosis. This model is useful to be built upon as new precise experimental data are emerging. The constraint-based modeling method can also be applied to quantitative modeling of other signaling pathways.

## Introduction

**Figure 1:**
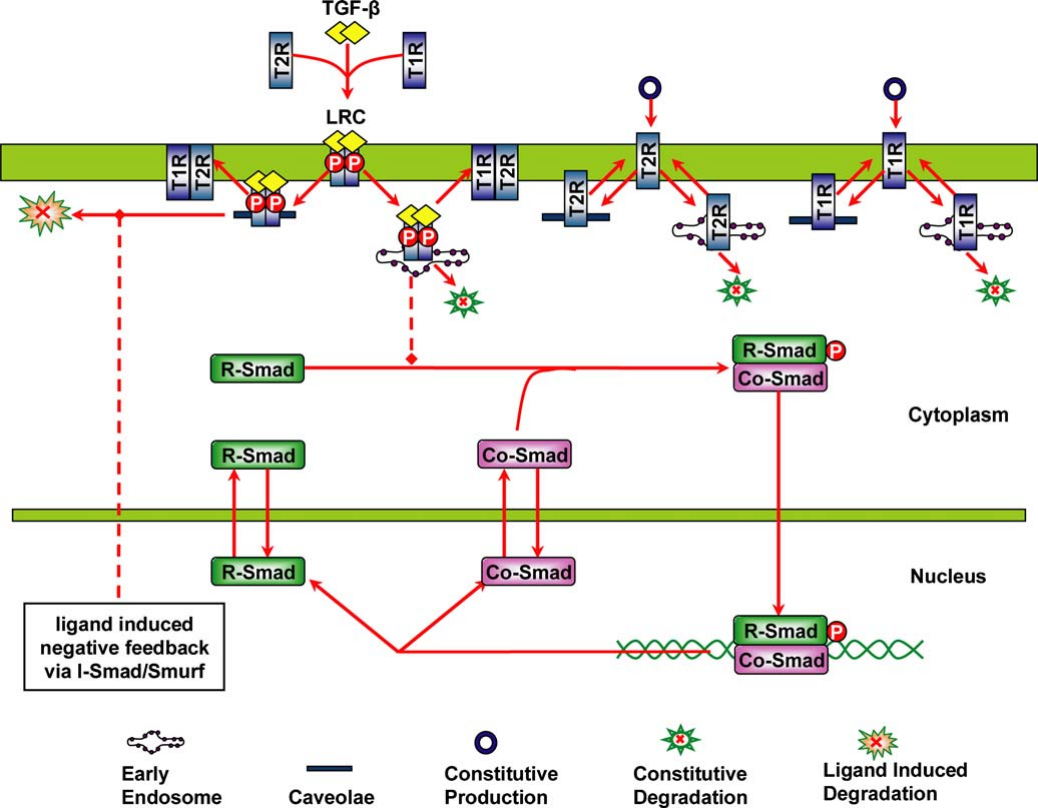
Schematic representation of Smad dependent TGF-β signaling pathway. Detailed information about this pathway is described in the text.

The transforming growth factor β (TGF-β) superfamily consists of TGF-βs, bone morphogenetic proteins (BMPs), activins and related proteins. These proteins regulate numerous cellular processes, such as cell proliferation, differentiation, apoptosis and specification of developmental fate [1], [2]. TGF-β initiates signaling by forming a ligand-receptor complex with the type I and type II receptors at cell surface. The activated receptor complex propagates the signal inside by phosphorylating the receptor-regulated Smad (R-Smad). Activated R-Smad then forms a heteromeric complex with common mediated Smad (Co-Smad), Smad4. These complexes accumulate in the nucleus and regulate gene expression in a cell-type-specific manner through interactions with transcription factors, coactivators and corepressors [3]. The nuclear Smad complexes are then dephosphorylated by Smad phosphatase [4]. Another important player is inhibitory Smad (I-Smad), Smad7, which recruits Smurf to the TGF-β receptor complex to facilitate the ubiquitin dependent degradation of receptors [5]. Both R-Smad and Smad4 continuously shuttle between the cytoplasm and nucleus in uninduced cells and also in presence of TGF-β signal [6]–[8]. The receptors and activated ligand-receptor complex are internalized through two distinct endocytic routes, the clathrin-dependent endocytosis and the caveolar lipid-raft mediated endocytosis [9], [10]. Figure 1 schematically depicts the Smad dependent TGF-β signaling pathways.

Quantitative modeling studies of signaling pathways have been successfully applied to understand complex cellular processes [11]–[14]. Traditionally, the quantitative models are validated by fitting western-blot data. Due to the complexity of the model and the quality of the data, over-fitting is a common problem for parameter estimation. When the model has many estimated parameters and the corresponding data is a few, the over-fitting problem might lead to some unwarranted conclusions because the parameter set in the model might be a special domain of possible parameter sets which can similarly fit the data well, but result in different predictions. On the other hand, genome-scale constraint-based models of metabolism have been constructed and used to successfully interpret and predict cellular behavior [15]. Constraint-based modeling is an effective method to narrow the range of the possible parameter space for quantitative models. However, there is less attention to apply the constraint-based modeling for the quantitative analysis of signaling pathways. Here, we proposed a constraint-based modeling method to build a comprehensive mathematical model for the Smad-dependent TGF-β signaling pathway by fitting experimental data and incorporating the qualitative constraints from the experimental analysis.

So far, several mathematical models have been proposed for the TGF-β signaling pathways. Vilar *et al.* proposed a concise model for the TGF-β receptor trafficking network [16]. On the other hand, Clarke *et al.* [17] and Melke *et al.* [18] proposed the models for the Smad nucleocytoplasmic shuttling and Smad phosphorylation response to the TGF-β signal without considering the receptor trafficking steps. In this study, we established a comprehensive mathematical model for the TGF-β signaling pathway, which includes the signal transduction, the receptor endocytosis, the Smad nucleocytoplasmic shuttling, and the ligand induced negative feedback. The simulation analysis of the model agrees well with the experimental analysis of TGF-β pathway, such as the time course of nuclear phosphorylated Smad, the subcellular location of Smad, and Smad phosphorylation response to different concentrations of TGF-β. We employed the mathematical model to study the dynamic relationship between receptor trafficking at the cell surface and the activation of the phosphorylated Smad in the nucleus. The simulation results indicate that the signal response to TGF-β is regulated by the balance between clathrin dependent endocytosis and non-clathrin mediated endocytosis.

## Materials and Methods

### Mathematical model of the Smad dependent TGF-β signaling pathway

We proposed here a comprehensive model for Smad dependent TGF-β signaling pathway in mammalian cells, which includes three modules: receptor trafficking and signaling; Smad nucleocytoplasmic shuttling and signaling and I-Smad negative regulation. In the module of receptor trafficking, the model takes into account the following essential elements: (i) constitutive receptor synthesis and degradation; (ii) receptor and ligand-receptor complex trafficking by two distinct endocytic routes; (iii) the distribution of receptors and ligand-receptor complex in different pools, such as membrane surface, clathrin-coated pit, early endosome and caveolar lipid-raft; (iv) the formation of activated receptor complex induced by TGF-β. In the module of Smad nucleocytoplasmic shuttling, the signal events we consider are (i) Smad nucleocytoplasmic shuttling; (ii) Smad complex formation; (iii) nuclear Smad complex dephosphorylation by nuclear phosphatase. In the module of I-Smad negative regulation, the negative feedback contributing to the ligand-induced degradation of the receptors by I-Smad is simplified and modeled as a black box from the nuclear phosphorylated Smad. We made the following assumptions for the model:

1. Experimental analyses indicate that TGF-β receptor trafficking is not affected by TGF-β stimulation [9], [10]. We assume that the corresponding internalization and recycling rates for type I receptor, type II receptor and activated ligand-receptors are the same.
2. Referring to the concise model of signal processing in the TGF-β ligand-receptor network [16], we assume that the rate of sequential formation of ligand-receptor complex is proportional to the amount of ligand, type I receptor and type II receptor.
3. The experimental data shown by Lin, *et al.* indicate that the variation of total amount of Smad is small [4]. We assume that the total amount of Smad is constant. Therefore, the production and degradation of Smad are not considered in this model.
4. Previous experimental analyses suggest that I-Smad/Smurf complex targets the lipid-raft-bound receptor for degradation which leads to the ligand-induced negative feedback [5], [9]. We assume that the rate of ligand-induced degradation of receptors is proportional to the amount of nuclear phoshporylated Smad and the receptor complex in caveolar lipid-raft.

In order to compare with the published data, we used Smad2 denoting R-Smad. We used the law of mass-action to describe the rate of signal transduction steps. The time-dependent changes of the concentrations of the signaling proteins and protein complexes are determined by the following system of differential equations:
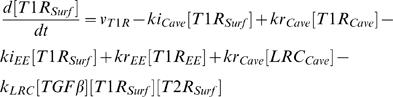
(1)

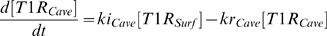
(2)

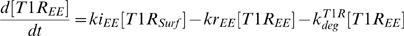
(3)

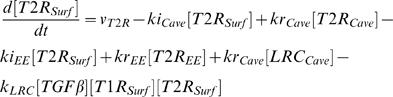
(4)

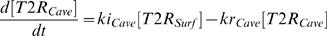
(5)

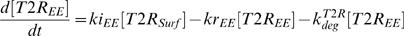
(6)

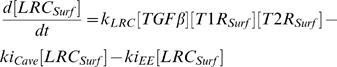
(7)

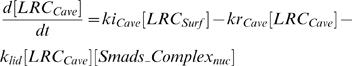
(8)

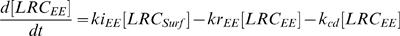
(9)

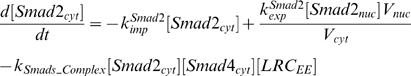
(10)

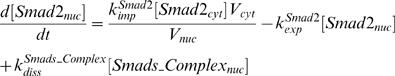
(11)

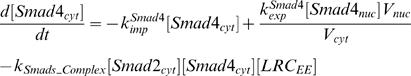
(12)

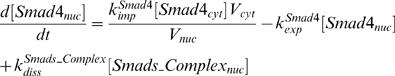
(13)

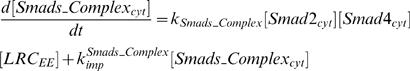
(14)

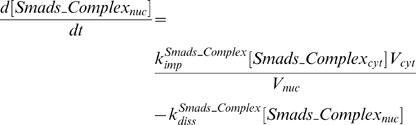
(15)

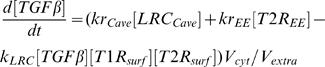
(16)The model is composed of an ordinary differential equations system with 16 state variables and 20 parameters. The values and the corresponding biological meaning of the parameters are listed in Table 1. The initial conditions and the biological meaning of the variables are listed in Table 2.

**Table 1:** Parameter values in the model

Parameter symbol	Biological meaning	Value	Unit	Reference
*v_TIR_*	type I receptor production rate constant	0.0103	nM/min	estimated
*v_T2R_*	type II receptor production rate constant	0.02869	nM/min	estimated
*ki_EE_*	internalization rate constant of receptor from cell surface to early endosome	0.33	min^−1^	[9], [16]
*kr_EE_*	recycling rate constant of receptor from early endosome to cell surface	0.033	min^−1^	[9], [16]
*ki_Cave_*	internalization rate constant of receptor from cell surface to caveolar lipid-raft	0.33	min^−1^	[9], [16]
*kr_Cave_*	recycling rate constant of receptor from caveolar lipid-raft to cell surface	0.03742	min^−1^	estimated
*k_cd_*	constitutive degradation rate constant for ligand-receptor complex in early endosome	0.005	min^−1^	[9]
*k_LRC_*	ligand-receptor complex formation rate constant from TGF-β and receptors	2197	nM^−2^min^−1^	estimated
*k_lid_*	ligand induced degradation rate constant for ligand-receptor complex in caveolar lipid-raft	0.02609	min^−1^	estimated
*k^T1R^_deg_*	constitutive degradation rate constant for type I receptor in early endosome	0.005	min^−1^	[21]
*k^T^* ^2*R*^ *_deg_*	constitutive degradation rate constant for type II receptor in early endosome	0.025	min^−1^	[21]
*k^Smad^* ^2^ *_imp_*	nuclear import rate constant for Smad2	0.16	min^−1^	[8]
*k^Smad^* ^2^ *_exp_*	nuclear export rate constant for Smad2	1	min^−1^	[8]
*k^Smad4^_imp_*	nuclear import rate constant for Smad4	0.08	min^−1^	[8]
*k^Smad4^_exp_*	nuclear export rate constant for Smad4	0.5	min^−1^	[8]
*k_Smads_Complex_*	formation rate constant for the phosphorylated Smad complex	6.85×10^−5^	nM^−2^min^−1^	estimated
*k^Smads_Complex^_imp_*	nuclear import rate constant for the phosphorylated Smad complex	0.16	min^−1^	[8], [22]
*k^Smads_Complex^_diss_*	dissociation rate constant for the nuclear phosphorylated Smad complex	0.1174	min^−1^	estimated
*V_cyt_/V_nuc_*	ratio of cytoplasmic to nuclear volume	3.0	3.0	[8]
*V_cyt_/V_extra_*	ratio of cytoplasmic volume to the average extracellular medium volume per cell	0.001	0.001	[19], [20]

**Table 2:** Initial conditions of state variables (proteins) in the model

Variable symbol	Biological meaning	Value of the initial condition
*T1R_surf_*	type I receptor at cell surface	0.237
*T1R_Cave_*	type I receptor in caveolar lipid-raft	2.092
*T1R_EE_*	type I receptor in early endosome	2.06
*T2R_surf_*	type II receptor at cell surface	0.202
*T2R_Cave_*	type II receptor in caveolar lipid-raft	1.778
*T2R_EE_*	type II receptor in early endosome	1.148
*LRC_Surf_*	ligand-receptor complex at cell surface	0
*LRC_Cave_*	ligand-receptor complex in caveolar lipid-raft	0
*LRC_EE_*	ligand-receptor complex in early endosome	0
*Smad2_cyt_*	Smad2 in the cytoplasm	492.61
*Smad2_nuc_*	Smad2 in the nucleus	236.45
*Smad4_cyt_*	Smad4 in the cytoplasm	1149.4
*Smad4_nuc_*	Smad4 in the nucleus	551.72
*Smads_Complex_cyt_*	phosphorylated Smad complex in the cytoplasm	0
*Smads_Complex_nuc_*	phosphorylated Smad complex in the nucleus	0
*TGFβ*	TGF-β in the extracellular medium	0.08 nM for 2 ng/ml

The initial conditions of the variables are set as the steady state concentration of the corresponding proteins in the uninduced cell which are derived from the parameter values described in Table 1.

### Derivation of the parameter values

In order to keep the consistency of the parameter values, we derived the parameter values from experimental analysis in epithelial cells. In particularly, most of the data used in this study is from experimental analysis of HaCaT (Human keratinocyte cell line) cells. The rational guided the derivation of relative parameter values is described in the following:

- **Parameters for the volume ratio of different compartments:** The model distinguishes the different compartments of extracellular medium, cytoplasm and nucleus where the signaling steps take place. The average cytoplasimc/nuclear volume ratio (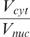) was measured as about 3 in reference [8]. The volume of HaCaT cell is estimated as about 1.4×10^−12^ Liter [19], [20], therefore, we can derive the volume of cytoplasm and nucleus are 1.05×10^−12^ Liter and 3.5×10^−13^ Liter, respectively. A typical cell culture experiment would have a cell density of 1.0×10^6^ cells/ml. The average extracellular medium volume per cell (*V_extra_*) is about 1.0×10^−9^ Liter. Hence, the volume ratio of cytoplasm to extracellular medium (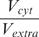) is set to a value of 0.001.
- **Rate constant of receptor constitutive degradation**: Pervious experimental data indicate that the type II receptor has a half-life of about 1 hr, whereas type I receptors are more stable, with a half-life of about 4–6 hr [21]. The receptors are constitutively degraded from early endosomes and about 50% percent of initial labeled receptors locate in early endosome [9]. Rescaling the degradation rate of the total receptors to the early endosome receptors results in constitutive degradation rate constant of type I receptor (*k^T1R^_deg_*) and type II receptor (*k^T^* ^2*R*^ *_deg_*) with value of 0.005 min^−1^ (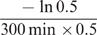) and 0.025 min^−1^ (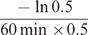), respectively. On the other hand, Figure 3 of reference [9] shows that only 30% of the initial labeled receptor complex remain in the cell after 8 h when the caveolar endocytosis is inhibited. Based on this information, we can derive the constitutive degradation rate constant of ligand-receptor complex (*k_cd_*) is 0.005 min^−1^ (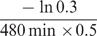).
- **Rate constant of receptor internalization and recycling**: Vilar *et al.* derived the rate constant of receptor internalization and recycling based on the experimental observations [16]. On the other hand, reference [9] indicate that receptors are internalized through clathrin and non-clathrin independent endocytosis route with similar rates. Therefore, we choose the internalization rate constant of receptors (*ki_EE_* and *ki_Cave_*) with the value of 0.33 min^−1^ which is estimated in reference [16]. The recycling rate constant (*kr_EE_*) for the receptors recycled from early endosome back to the cell surface is set to a value of 0.033 min^−1^, which is derived in reference [16].
- **Rate constant for Smad nuclear import and export**: The nuclear import rate of Smad2 (*k^Smad^* ^2^ *_imp_*) was experimentally measured with the value of 0.16 min^−1^ in reference [8]. Reference [8] also measured that the ratio of mean nuclear fluorescence to mean cytoplasmic fluorescence (R) of Smad2 in uninduced cells is about 0.5. The R ratio of Smad2 is equivalent to the ratio of nuclear to cytoplasmic Smad2 concentrations. We can derive 
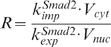
 according to equation (31). Therefore, the value for the nuclear export rate constant (*k^Smad^* ^2^ *_exp_*) in our model is about 1 min^−1^ (
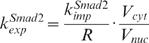
). On the other hand, Figure 5A of reference [8] also shows that the nuclear export rate of Smad4 is about half of the nuclear export rate of Smad2. Hence, the nuclear export rate constant of Smad4 (*k^Smad^* ^4^ *_exp_*) is 0.5 min^−1^. Since the R ratio of Smad4 is measured with a value of about 0.5 [8], we can derive the nuclear import rate constant of Smad4 (*k^Smad^* ^4^ *_imp_*) a value of 0.08 min^−1^.
- **Nuclear import rate constant of phosphorylated Smad:** Figure 3 of reference [22] shows that nuclear import rate between phoshporylated and unphosphorylated Smad2 is similar. Therefore, we set the import rate constant for the phosphorylated Smad complex (*k^Smads_Complex^_imp_*) to the same value as the import rate constant for unphosphorylated Smad2, which is 0.16 min^−1^.

There are 7 unknown parameter values in the model that are required to be estimated, i.e. *v_T1R_*, *v_T2R_*, *kr_Cave_*, *k_LRC_*, *k_lid_*, *k_Smads_Complex_* and *k^Smads_Complex^_diss_*.

### Steady state analysis of the model for the uninduced cell

We performed steady state analysis of the model for the uninduced cell without TGF-β stimulation. When there is no TGF-β present to the cells, the concentrations of the ligand-receptor complex and phosphorylated Smad complex are assumed to be zero.

For type I receptor, we can derive the following algebraic equations for the steady state of the unindcued cell:
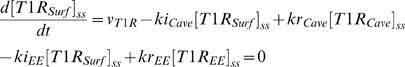
(17)

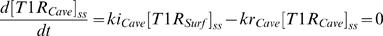
(18)

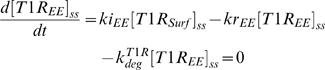
(19)The steady state concentrations of type I receptor at cell surface, in the lipid-raft and in the early endosome, obtained by solving the systems of algebraic equations (17–19), are
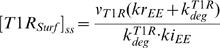
(20)

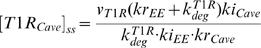
(21)

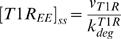
(22)The steady state concentrations of type II receptor at cell surface, in the lipid-raft and in the early endosome are
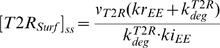
(23)

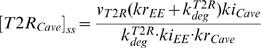
(24)

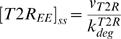
(25)For the Smad2 in the cytoplasm and nucleus, the following algebraic equations should be satisfied for the steady state of the unindcued cell:
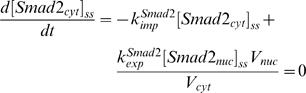
(26)

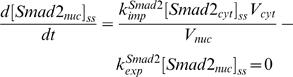
(27)Because the total amount of Smad2 is a constant, mass conservation reads: 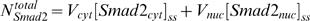
(28)Solving the algebraic equations (26–28) yields the steady state concentrations of cytoplasmic and nuclear Smad2 in the uninduced cell as following:
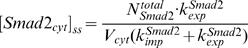
(29)

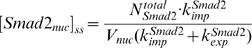
(30)The ratio of nuclear to cytoplasmic Smad2 concentration corresponds to the ratio of mean nuclear fluorescence to mean cytoplasmic fluorescence (R) of Smad2, according to equations (29–30), which is
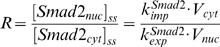
(31)The steady state concentrations of cytoplasmic and nuclear Smad4 in the uninduced cell are
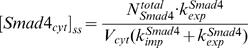
(32)

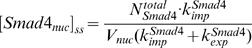
(33)The total amount of Smad2 (*N^total^_Smad_*_2_) and Smad4 (*N^total^_Smad4_*) are estimated to be 6×10^−19^ mol per cell and 1.4×10^−18^ mol per cell in HaCaT cells, which corresponds to about 3.6×10^5^ and 8.4×10^5^ molecules per cell, respectively [23].

Without the treatment of TGF-β, the concentrations of signaling proteins will arrive at steady state balancing protein production, degradation, receptor endocytosis and Smad protein nucleocytoplasmic shuttling. We set the initial concentrations of the signaling proteins as their steady state values before TGF-β is added.

### Parameter estimation by constraint-based modeling

The parameter estimation for the 7 unknown values of rate constants was done using a modified version of the tool SBML-PET [24], which incorporates stochastic ranking evolution strategy (SRES) for parameter estimation jobs. SRES is an evolutionary optimization algorithm that uses stochastic ranking as the constraint handling technique [25]. The objective of the parameter estimation is to find the most feasible parameters in the model that reproduce the quantitative experimental data for the TGF-β signaling pathway. At the same time, the model with the estimated parameters should satisfy some qualitative experimental observation of this pathway. Therefore, the corresponding quantitative time course data is used in the objective function definition and the qualitative data is coded as constraints during the optimization process. We used two time courses of Smad phosphorylation for parameter estimation:

1. The quantitative western blot data reported in Figure 1A in Lin *et al.* [4] gives us the time course of Smad2 in the whole HaCaT cell extract in the presence of continuous TGF-β stimulation for 24 hours. We normalized the data to its maximum value which gives us the dynamic change of the protein. We also normalized corresponding simulation data to its maximum value so that we can compare the experimental data and the simulation data.
2. The quantitative western blot data reported in Figure 1C in Lin *et al.* [4] gives us the time course of Smad2 phosphorylation under a different condition. TGF-β (2 ng/ml) is added at first 30 minutes, then washed out and type I receptor kinase inhibitor SB431542 is added to terminate the signal.

Other qualitative information from the literatures is used as constraints encoded within SRES method.

1. The number of total receptors per cell falls into the range of 1000 to 100000 per cell according to the distribution of TGF-β receptors in a wide spectrum of cell types reported in Wakefield *et al.* [26].
2. Experimental results of receptor distribution at the cell surface, in the early endosomes and caveolin-positive vesicles indicate that about 40∼50% percent of total receptors are located in the early endosomes [9], [10]. We set a constraint for the distribution of receptors, that the about at least 30% and at maximum 60% of receptors locate in the early endosome.
3. Schmierer *et al.* quantified the Smad redistribution in HaCaT cells upon 2 ng/ml TGF-β with photobleaching experiments using EGFP-Smad (enhanced green fluorescent protein) fusions [8]. The study indicates that the ratio of nuclear to cytoplasmic Smad2 concentrations is about 2.5 after 1 hour TGF-β treatment. After TGF-β stimulus, about 80% of the Smad2 in the nuclues is phosphorylated or complexed. We set constraints for the corresponding Smad2 distribution: the ratio of nuclear to cytoplasmic Smad2 concentrations should be in the range of 1.5∼3.5 and 60%∼90% of the Smad2 in the nucleus is phosphorylated or complexed after 1 hour TGF-β treatment.
4. Previous experimental data indicate that 0.5 ng/ml (20 pM) of TGF-β is sufficient to yield a maximal response of Smad phosphorylation after 1 hour TGF-β treatment [27], [28]. We encode this information as a constraint that the ratio of Smad phosphorylation level with the dose of 0.5 ng/ml to that with the dose of 2 ng/ml TGF-β at 60th minute should be larger than 0.9 and smaller than 1.1.

## Results and Discussion

### Comparison of kinetic simulation with experimental analysis

**Figure 2:**
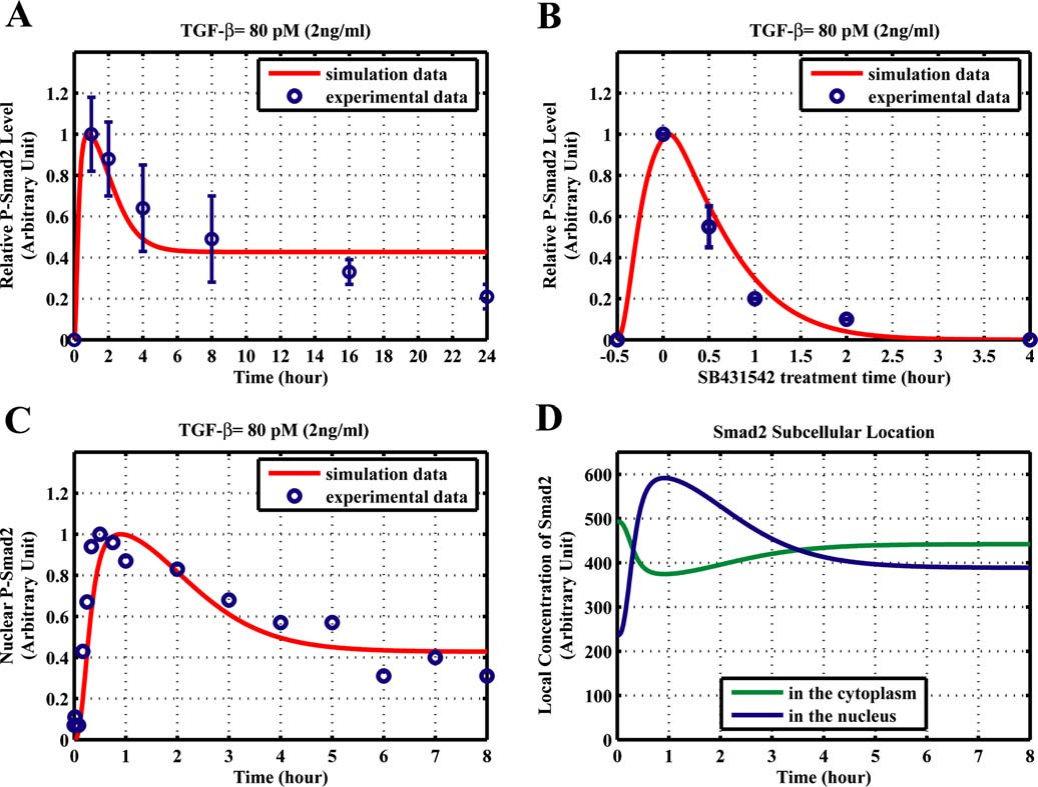
Comparison of experimental analysis and simulation results from the model obtained by constraint-based modeling method. (A–B) for “in-sample fit”. (C–D) for “out-sample fit”. (A) Comparison of the model time course and experimental time course of Smad2 phosphorylation with 24 hours TGF-β treatment. The experimental data is normalized from Figure 1A in Lin *et al.* [4]. (B) Effect of type I receptor kinase inhibitor SB431542. Cells were treated with TGF-β for 30 minutes, then were washed out TGF-β at 30th minute and added SB431542 to prevent rephosphorylation of Smad2. The experimental data is normalized from Figure 1C in Lin *et al.* [4]. (C) Comparison of the model time course with an experimental time course of nuclear phosphorylated Smad2 after TGF-β treatment (80 pM, 2 ng/ml). The western-blot data reported by Inman *et al.* (Fig. 1A, top panel) is quantified with Scion Image software [29]. (D) Subcellular location of Smad2 after TGF-β treatment (80 pM). The concentrations shown here refer to the local concentrations in cytoplasm and nucleus.

We first check whether the model obtained by constraint-based modeling method can reproduce the experimental data used for parameter estimation, which can give us the information about quality of “in-sample fit” (how good is the model fitting the data used for parameter estimation). Experiments reported that the signal would peak at about 30–60 min after TGF-β addition. The result shown in Figure 2A indicates that the simulated time courses of Smad2 phosphorylation agree well with the experimental data [4]. The model is also able to reproduce the experimental observation that the treatment with type I receptor kinase inhibitor SB431542 will cause rapid decrease of the nuclear phosphorylated Smad2 level [4], [29] (Figure 2B). Taken these simulation results together, the model has good “in-sample fit” for the data.

**Figure 3:**
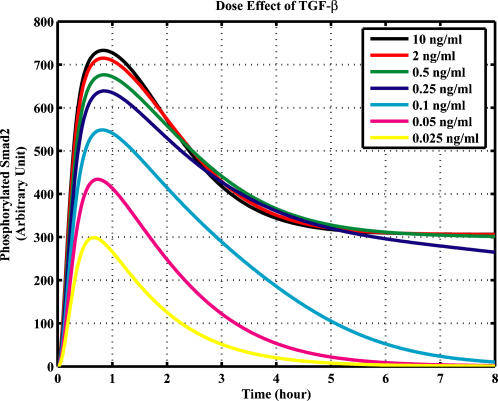
Effects on Smad2 phosphorylation by different doses of TGF-β.

We next asked whether the model has a good match to other experimental data that were not used for parameter estimation. This test can be regarded as “out-sample fit” or model validation. The result shown in Figure 2C indicates that the simulated time courses of nuclear phosphorylated Smad2 agree well with the experimental data [29]. As a further test, we calculated the subcellular location of Smad2 from the simulation result of the model. The results are in agreement with previous reports that TGF-β causes a change in the overall Smad2 distribution from predominantly cytoplasmic to predominantly nuclear [6]–[8], [29]. After TGF-β treatment, Smad2 proteins are rapidly accumulated in the nucleus and then return to the cytoplasm (Figure 2D). Finally, we tested whether the model can predict well the signal response to different dose of TGF-β. Previous experimental data indicate that the response of Smad phosphorylation after 1 hour TGF-β treatment will be saturated when the concentration of TGF-β is larger than 0.5 ng/ml [27], [28]. The model successfully predicts the dose-response of Smad phosphorylation upon different concentrations of TGF-β (Figure 3).

### Model performance is significantly improved by constraint-based modeling

**Figure 4:**
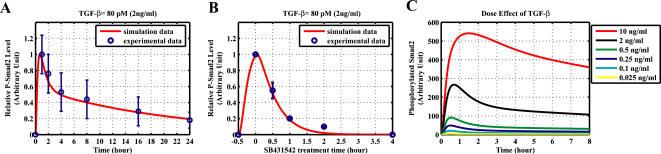
Comparison of experimental analysis and simulation results from the model obtained by only fitting the time course data. (A–B) The model has been over-fitted for “in-sample fit”. (C) The model has a bad prediction for “out-sample fit”. (A) Comparison of the model time course and experimental time course of Smad2 phosphorylation with 24 hours TGF-β treatment. Experimental data is the same as described in Figure 2A. (B) Effect of type I receptor kinase inhibitor SB431542. Experimental data is the same as described in Figure 2B. (C) Effects on Smad2 phosphorylation by different doses of TGF-β.

We compared the performance of the model generated by constraint-based modeling method and that of the model obtained by only fitting the time course data. We first compared the experimental data and simulation results from the model obtained by only fitting the time course data. The results shown in Figure 4 indicate that the model has been over-fitted to the data used for parameter estimation (Figure 4A–4B), but it has bad predictions for the data that are not used for parameter estimation. For example, experimental evidence shows that the response of Smad phosphorylation after 1 hour TGF-β treatment will be saturated when the concentration of TGF-β is larger than 0.5 ng/ml [27], [28]. However, the predicted time course of phosphorylated Smad2 in the model, obtained by only fitting the time course data, is not saturated even for a very high dose of TGF-β (10 ng/ml), which contradicts the experimental results (Figure 4C).

On the other hand, we compared the possible variation of parameter sets of the models by these two different methods. For each method, we independently generated 1000 parameter sets which make the model have similar goodness of fitting the time course data of Smad phosphorylation (Table S1 and Table S2). According to the statistical result for the parameter sets shown in Table 3, the ranges of the variation for the 7 estimated parameter values are significantly narrowed by constraint-based modeling method.

**Table 3:** Range of the variation for the estimated parameters in the 1000 parameter sets

	Obtained by constraint-based modeling method			Obtained by only fitting the time course data		
Parameter	Minimum value	Maximum value	Range of variation: log_10_(max/min)	Minimum value	Maximum value	Range of variation: log_10_(max/min)
*v_T1R_*	0.007482	0.02712	0.55927	0.001309	0.5195	2.5986
*v_T2R_*	0.02167	0.1101	0.70593	0.008435	4.874	2.7618
*kr_Cave_*	0.03737	0.04243	0.05515	0.001571	0.04571	1.4638
*k_LRC_*	1305	86850	1.8232	0.1088	6.835e+006	7.7981
*k_lid_*	0.001011	0.9774	2.9853	1.383e-005	0.9988	4.8587
*k_Smads_Complex_*	2.283×10^−5^	9.694×10^−5^	0.628	3.152e-007	0.000203	2.8089
*k^Smads_Complex^_diss_*	0.09519	0.1298	0.13468	0.07243	0.9985	1.1394

The values of the 1000 parameter sets obtained by each method are available in Table S1 and Table S2

**Figure 5:**
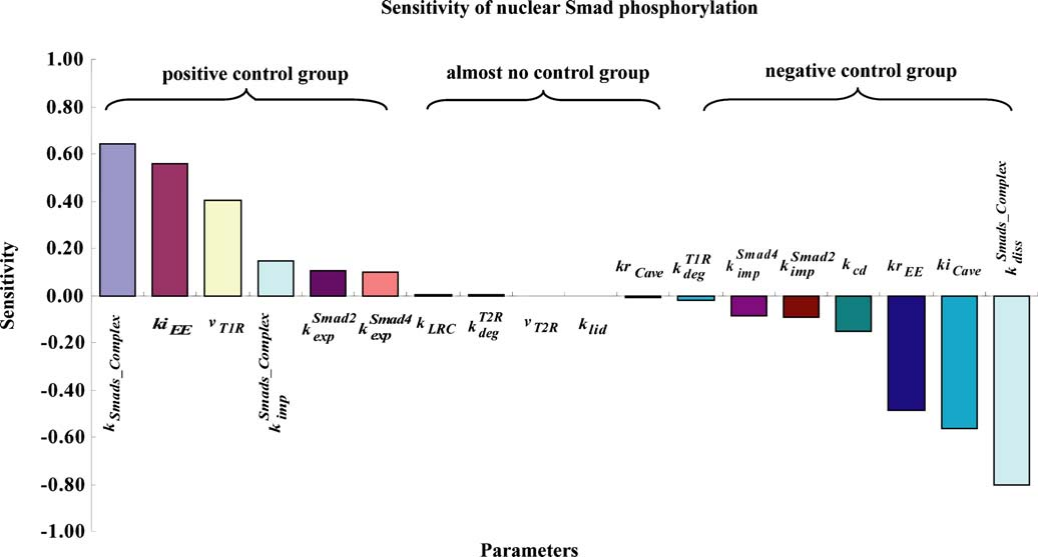
Sensitivity analysis of the rate constants on nuclear Smad phosphorylation. The original values of the sensitivities are present Table S3

Previous modeling studies of TGF-β signaling pathway indicate that the Smad phosphorylation response to TGF-β is robust to a large variation of some parameters [17], [18]. The large variations in the estimated parameter sets are usually caused by two reasons. The data quality is the main reason for the large variation of parameter sets obtained by only fitting the time course data. When we fit the western-blot data, we actually fit the scaled data. However, the parameter for the scaled coefficient is unknown, which can vary in a large range if we only fitting the scaled time course data without considering other qualitative constraints. Another reason comes from the insensitivity of some parameters to the signal response. For example, the sensitivities of the parameters *k_LRC_*, *v_T2R_* and *k_lid_* are very small (Figure 5), which means the output of the model (Smad phosphorylation level) is robust to the variation of these insensitive parameters.

### Sensitivity analysis of the model

We next systematically investigated the sensitivity of all the rate constants and found those whose perturbation the pathway is most sensitive or most robust against. Response sensitivities were used for quantifying the effects of all the rate constants on the concentration of signaling proteins. In this model, we regard nuclear phosphorylated Smad complex (nuclear phosphorylated Smad2) as the readout of the signal in this pathway because experimental results indicate that nuclear phosphorylated Smad complex acts as transcription factor to induce the expression of many genes [1], [3]. We determined the sensitivity of the integral concentrations of the nuclear phosphorylated Smad complex from the beginning of the TGF-β activation to the end of the simulation time (8 hours). The definition of response sensitivity for nuclear phosphorylated Smad is as following:
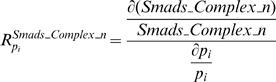
(34)As shown in Figure 5, we can divide the parameters into three groups: positive control, negative control and almost no control to the changes of nuclear Smad complex. The strong positive and negative control groups belong to the reactions participating phosphorylation of Smad, receptor endocytosis, type I receptor production. The most negative control on the concentration of nuclear Smad complex is the rate constants corresponding to the dephosphorylation of nuclear Smad complex, which implies that the nuclear phosphatase has a strong negative control on the nuclear phosphorylated Smad level. Finally, we want to point out that all these analyses are based on small perturbations of relative parameters according to the definition of response coefficient.

### The regulation of the signal: balance between clathrin dependent endocytosis and non-clathrin mediated endocytosis

**Figure 6:**
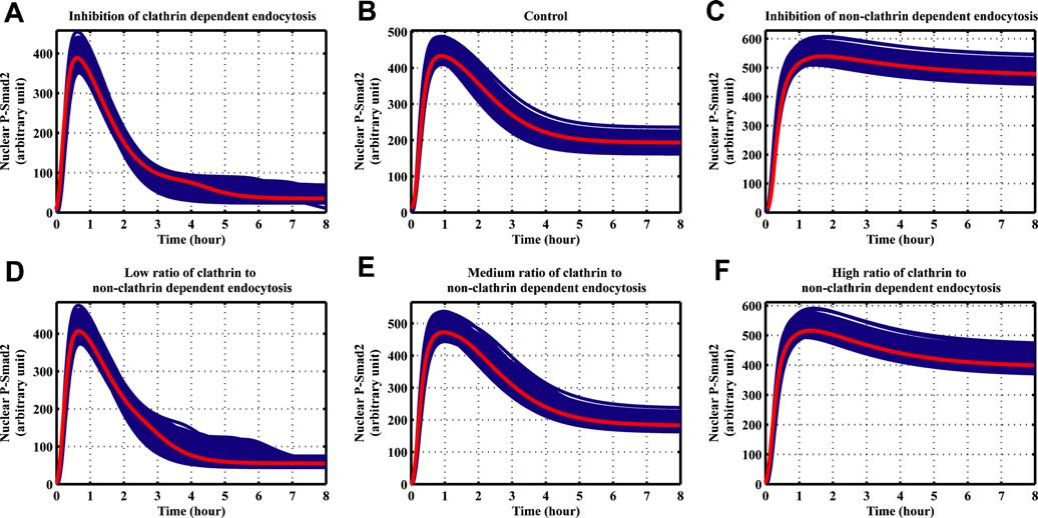
Computational simulations of the time course of nuclear phosphorylated Smad2 by the inhibition of different receptor endocytosis in 1000 parameter sets estimated by constraint-based modeling method. The red lines refer the simulations for the parameter values listed in Table 1. Blue lines correspond to the 1000 parameter sets with the estimated parameter values listed in the Table S1. (A) Same parameter values as those in parameter sets with the exception that clathrin dependent internalization rate constant is decreased by a factor of 10: *ki_EE_* = 0.033 min^−1^. (B) Same parameters values as those in parameter sets. (C) Same parameter values as those in parameter sets with the exception that non-clathrin dependent internalization rate constant is decreased by a factor of 10: *ki_Cave_* = 0.033 min^−1^. (D) Same parameter values as those in parameter sets with the exception that *ki_EE_* is decreased by a factor of 10 and *ki_Cave_* is decreased by a factor of 2: *ki_EE_* = 0.033 min^−1^, *ki_Cave_* = 0.165 min^−1^. (E) Same parameter values as those in parameter sets with the exception that *ki_EE_* and *ki_Cave_* are decreased by a factor of 10: *ki_EE_* = 0.033 min^−1^, *ki_Cave_* = 0.033 min^−1^. (F) Same parameter values as those in parameter sets with the exception that *ki_EE_* is decreased by a factor of 2 and *ki_Cave_* is decreased by a factor of 10: *ki_EE_* = 0.165 min^−1^, *ki_Cave_* = 0.033 min^−1^.

In the experimental studies, potassium depletion can be used to interfere with clathrin-dependent trafficking of receptors, which can inhibit the TGF-β signal [9], [30]. On the other hand, nystatin treatment causes the inhibition of the non-clathrin endocytosis pathway [9]. The simulation analysis of the model indicates that the inhibition of clathrin dependent endocytosis causes a transient response of Smad2 phosphorylation (Figure 6A). The simulation result also shows that inhibition of non-clathrin dependent endocytosis increases TGF-β signal amplitude, which produces a prolonged signal (Figure 6C).

What will happen if both clathrin dependent and non-clathrin dependent endocytosis are inhibited? The simulation results indicate that the key quantity is the ratio of clathrin to non-clathrin dependent endocytosis rate. A transient response of Smad2 phosphorylation appears upon the combination of a strong inhibition of clathrin dependent endocytosis and a weak inhibition of non-clathrin mediated endocytosis, which corresponds to a low ratio of clathrin to non-clathrin dependent endocytosis rate (Figure 6D). Furthermore, when both clathrin dependent endocytosis and non-clathrin dependent endocytosis are equally inhibited (a medium ratio of clathrin to non-clathrin dependent endocytosis rate), the signal response of Smad2 phosphorylation is similar as the control (Figure 6E). Finally, a prolonged signal response of Smad2 phosphorylation is observed (Figure 6F) with the combination of a weak inhibition of clathrin dependent endocytosis and a strong non-clathrin dependent endocytosis (a high ratio of clathrin to non-clathrin dependent endocytosis rate). Therefore, the TGF-β signal response is regulated by the balance between the strength of signal initiation from clathrin dependent endocytosis and the strength of negative feedback in the venue of non-clathrin mediated endocytosis.

Recently Vilar *et al.* proposed a concise computational model of signal processing in TGF-β superfamily ligand-receptor network [16]. This work indicates that the key quantity that determines the qualitative behavior of the pathway is the ratio of the constitutive to the ligand-induced rate of receptor degradation (CIR, constitutive-to-induced degradation ratio). Low CIR causes a transient increase of signal activity that returns to pre-stimulus levels. On the contrast, high CIR produces a permanently elevated level of signal activity. The concept of CIR refers to the rates of two degradations process rather than the simple expression of the degrading rate constants. This conclusion is affirmed by our analysis of the balance between clathrin dependent endocytosis versus non-clathrin mediated endocytosis. Our model shows the TGF-β signal response is regulated by the ratio of clathrin to non-clathrin endocytosis rate. On the other hand, the concise model doesn't distinguish the receptors in early endosomes and in caveolar lipid-raft. For ligand-induced degradation of receptors, the concise model simply regards the processes of the non-clathrin dependent internalization, recycling and the degradation of the receptors in caveolar lipid-raft as one-step of ligand induced receptor degradation at cell surface. Therefore, the control role of CIR ratio proposed in the concise model is consistent with regulation role of the clathrin to non-clathrin endocytosis rate ratio in the comprehensive model of this work.

## Supporting Information

Table S1 (0.19 MB XLS)

Table S2 (0.19 MB XLS)

Table S3 (0.12 MB XLS)
